# Association between brain health outcomes and metabolic risk factors in persons with diabetes

**DOI:** 10.1002/acn3.51859

**Published:** 2023-07-30

**Authors:** Evan L. Reynolds, Kristen Votruba, Clifford R. Jack, Richard Beare, Robert I. Reid, Gregory M. Preboske, Camille Waseta, Rodica Pop‐Busui, Robert G. Nelson, Brian C. Callaghan, Eva L. Feldman

**Affiliations:** ^1^ Department of Neurology University of Michigan Ann Arbor Michigan USA; ^2^ Department of Psychiatry University of Michigan Ann Arbor Michigan USA; ^3^ Department of Radiology Mayo Clinic Rochester Minnesota USA; ^4^ Peninsula Clinical School Monash University Melbourne Victoria Australia; ^5^ Chronic Kidney Disease Section National Institute of Diabetes and Digestive and Kidney Diseases Phoenix Arizona USA; ^6^ Department of Internal Medicine, Division of Metabolism, Endocrinology and Diabetes University of Michigan Ann Arbor Michigan USA

## Abstract

We performed a cross‐sectional study to determine associations between cognition and MRI‐derived brain outcomes, with obesity, diabetes duration, and metabolic risk factors in 51 Pima American Indians with longstanding type 2 diabetes (T2d) (mean [SD] age: 48.4 [11.3] years, T2d duration: 20.1 [9.1] years). Participants had similar cognition (NIH Toolbox Cognition Battery composite: 45.3 [9.8], *p* = 0.64, *n* = 51) compared to normative data. T2d duration, but not other metabolic risk factors, associated with decreased cortical thickness (Point Estimate (PE): −0.0061, 95%CI: −0.0113, −0.0009, *n* = 45), gray matter volume (PE: −830.39, 95%CI: −1503.14, −157.64, *n* = 45), and increased white matter hyperintensity volume (PE: 0.0389, 95%CI: 0.0049, 0.0729, *n* = 45).

## Introduction

Dementia is a global epidemic with 57 million estimated worldwide cases in 2019, a number expected to increase to 152 million by 2050.^1^ This increase may be partly attributed to the increasing worldwide prevalence of type 2 diabetes (T2d) and obesity,^2^, ^3^ as meta‐analyses suggest diabetes and midlife obesity associate with cognitive impairment and dementia.^4^, ^5^ Furthermore, our recent large, nationally representative study found diabetes complications associated with cognitive disorders.^6^

Pima American Indians have a higher prevalence of T2d with an earlier onset than individuals with T2d in the general population.^7^ Pima American Indians also have a higher prevalence of neuropathy, obesity, and hypercholesterolemia, as well as a higher incidence of chronic kidney disease (CKD).^8^, ^9^, ^10^, ^11^ Despite the early onset of T2d, and presence of both metabolic risk factors and diabetes complications, the effect on cognition, and functional/structural brain metrics has not been assessed in this population.

Additional evidence is needed to determine whether sensitive measurements of cognition and functional/structural brain metrics earlier in the lifespan associate with T2d, obesity, and other metabolic risk factors, particularly in a cohort with longstanding T2d. We determined the association between cognition and functional/structural brain metrics with T2d duration, obesity, other metabolic risk factors, neuropathy, cardiovascular autonomic neuropathy (CAN), and CKD in a cohort of Pima American Indians with longstanding T2d.

## Methods

### Population and recruitment

From 21 November 2017 to 2 July 2019, 51 Pima American Indians with T2d from the Gila River Community underwent metabolic phenotyping and cognitive assessments, with 45 also completing functional/structural MRI sequences. Inclusion/exclusion criteria were as previously described.^11^

### Cognition

The primary cognition measure was the NIH Toolbox Cognition Battery (NIHTB‐CB) fluid composite score, which aggregates five tests: flanker inhibitory control and attention (attention/executive function), picture sequence memory (episodic memory), list sorting (working memory), pattern comparison (processing speed), and dimensional change card sort (executive function). Secondary cognition measures included the above individual tests, picture vocabulary test (language) and oral reading recognition test (language). NIHTB‐CB outcomes were summarized as *t*‐scores with a mean = 50 and standard deviation (SD) = 10 based on participant age, sex, race, and education level.

### Functional/structural brain metrics

Acquired MRIs (Supplemental File) were analyzed by FreeSurfer software to measure brain morphometrics including white and gray matter volumes (unit = mm^3^), cortical thickness (unit = mm),^12^ and volume of white matter hyperintensities (WMH, unit = mm^3^) across 10 regions according to a previously published protocol.^13^ Head motion, eddy current distortion, Gibbs ringing, and Rician bias of the diffusion scans were corrected before fitting of the diffusion tensors as previously described.^14^ We calculated the global weighted average mean diffusivity(unit = mm^2^/s, higher in regions of tissue/membrane breakdown) and fractional anisotropy(range 0–1, marker of white matter microstructure coherence and/or myelination) across 115 regions (mean value across regions, weighted by number of voxels per region). Lastly, arterial spin labelling was performed, and the median cerebral blood flow (unit = mL/min/100 g of tissue) was averaged across 22 regions, as previously described.^15^

### Metabolic phenotyping

Height, weight, blood pressure, HbA1c, and a lipid panel were measured after overnight fasting. Diabetes onset was based on serial glucose tolerance testing and review of clinical records.

### Diabetes complications

Neuropathy was evaluated by the Michigan Neuropathy Screening Instrument (MNSI) combined index, CAN by the expiration/inspiration (E/I) ratio, and CKD by the glomerular filtration rate (GFR) (unit = mL/min), measured by the urinary clearance of iothalamate.

### Statistical analysis

Descriptive statistics characterized the study population. *Z*‐tests compared NIHTB‐CB outcomes to demographic‐adjusted normative data (mean = 50, SD = 10). Two‐sample *t*‐tests compared outcomes between participants with and without obesity. Pearson's correlation coefficients determined correlation between T2d duration and each outcome. Multivariable linear regression models were fit for each outcome separately to determine associations to individual metabolic risk factors. Models for functional/structural brain outcomes adjusted for the estimated total intracranial volume. Study outcomes included the NIHTB‐CB composite score, white matter volume, cortical thickness, gray matter volume, subcortical gray matter volume, total WMH volume, mean diffusivity, fractional anisotropy, and median cerebral blood flow. Given the number of outcomes, analyses were exploratory, and considered hypothesis‐generating.

**Figure 1:**
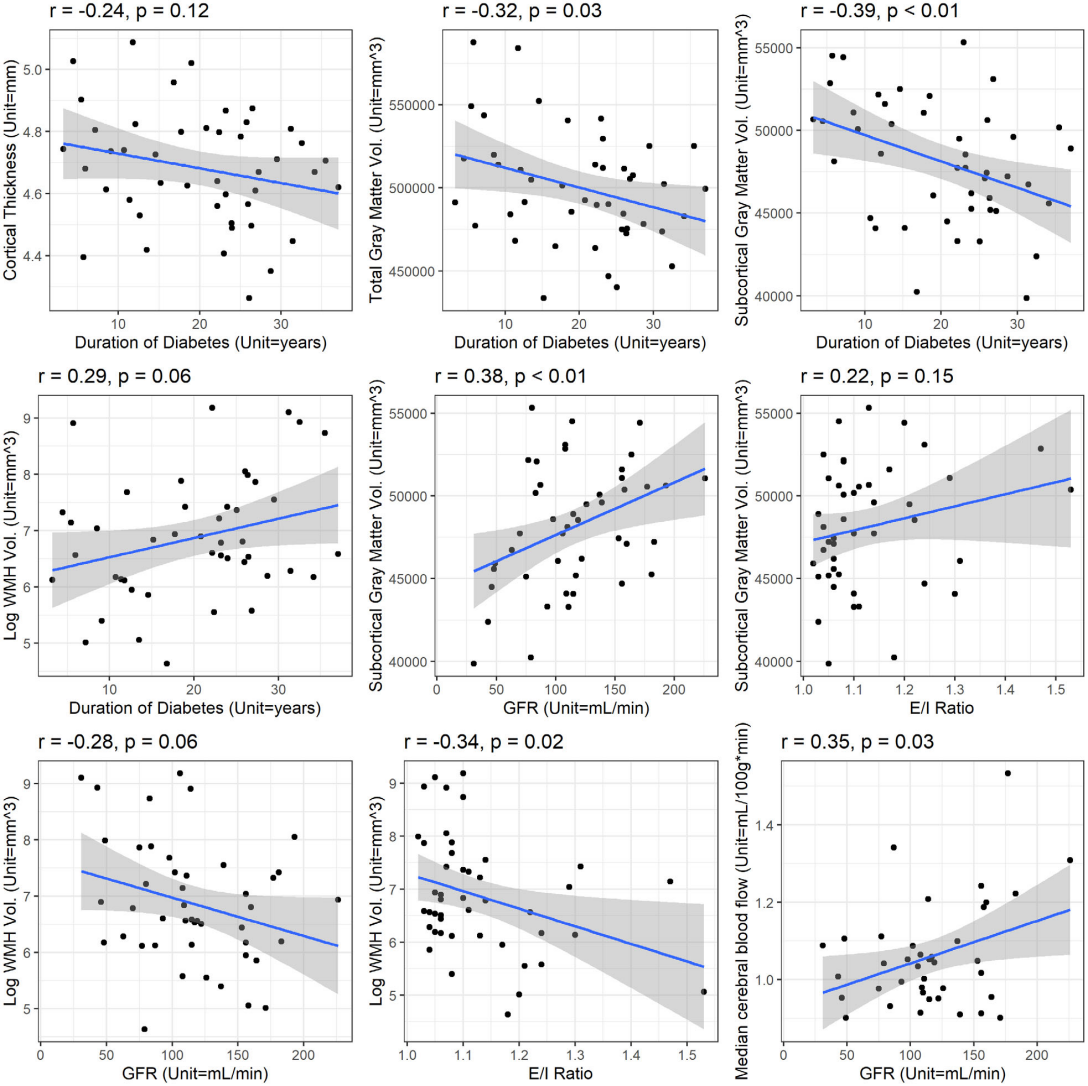
Correlation between T2d duration and diabetes complications with MRI‐derived structural and functional morphology. Fitted line represents results from a univariate linear regression model. Shaded area represents 95% confidence interval for predictions from the univariate linear regression model. E/I ratio, expiration/inspiration ratio; GFR, glomerular filtration rate; *p*, *p*‐value from Pearson's correlation coefficient; *r*, Pearson's correlation coefficient; vol., volume; WMH, white matter hyperintensities.

## Results

For all 51 participants, mean (SD) age was 48.4 (11.3) years, mean (SD) T2d duration was 20.1 (9.1) years, 74.5% were obese, and 74.5% were female (Table 1).

**Table 1:** Demographic information and metabolic risk factors of study participants.

Variable	Mean (SD) or *n* (%) (*n* = 51)
Age (years)	48.4 (11.3)
Sex (Female)	38 (74.5%)
T2d duration (years)	20.1 (9.1)
Height (cm)	163.9 (8.7)
Weight (kg)	93.9 (22.8)
BMI (kg/m^2^)	34.9 (7.7)
Systolic blood pressure (mmHg)	119 (17)
Diastolic blood pressure (mmHg)	72 (9)
HbA1c (%)	9.6 (2.3)
Triglycerides (mg/dL)	166 (111)
HDL (mg/dL)	42 (10)
LDL (mg/dL)	78 (41)
Total cholesterol (mg/dL)	151 (52)
Receiving medication for hypertension	30 (58.8%)

Analyses were completed using R.v.4.1.1.

BMI, body mass index; HbA1c, hemoglobin A1c; HDL, high‐density lipoproteins; LDL, low‐density lipoproteins.

Primary (*Composite Score*: 45.3 (9.8), *p* = 0.64) and secondary NIHTB‐CB cognition outcomes did not significantly differ from demographic‐adjusted normative data (Table 2). Participants had lowest cognitive scores on attention and executive functioning assessments (*Flanker Test*: 40.0 (6.3), *p* = 0.32). Cognition did not significantly differ between participants with and without obesity (*p* > 0.05). Longer T2d duration associated with poorer demographic‐adjusted scores on the picture sequence memory test (*r* = −0.29, *p* = 0.04), but not with any other NIHTB‐CB outcomes.

**Table 2:** Association between cognition, MRI‐derived structural and functional morphology, obesity, and T2d duration.

Variable	Overall (*n* = 51 cognition; *n* = 45 MRI)	*p*‐value: Pima American Indian cohort versus normative data (mean = 50, SD = 10)	Lean (BMI < 30) (*n* = 13 cognition; *n* = 12 MRI)	Obese (BMI ≥ 30) (*n* = 38 cognition; *n* = 33 MRI)	Class 1 obesity (30 ≤ BMI < 35) (*n* = 17 cognition; *n* = 16 MRI)	Class 2 obesity (35 ≤ BMI < 40) (*n* = 10 cognition; *n* = 7 MRI)	Class 3 (“Severe”) obese (BMI ≥ 40) (*n* = 11 cognition; *n* = 10 MRI)	*p*‐value: Obese versus nonobese	Correlation with T2d duration	*p*‐value: Correlation with T2d duration
Cognition										
Composite score (*n* = 51)	45.3 (9.8)	0.64	43.4 (9.3)	45.9 (10.1)	45.8 (10.1)	46.5 (12.1)	45.5 (8.8)	0.42	−0.01	0.95
Picture vocabulary test (*n* = 51)	48.9 (11)	0.91	49.8 (10.6)	48.6 (11.3)	46.6 (13.7)	49.4 (6.6)	51 (11)	0.74	−0.03	0.86
Picture sequence memory test (*n* = 51)	47.3 (7.1)	0.79	47.1 (7.6)	47.3 (7)	44.9 (8)	48.8 (5.1)	49.6 (6.2)	0.92	−0.29	0.04
Pattern comparison processing speed test (*n* = 51)	47.6 (13.9)	0.81	43.5 (11.2)	49 (14.6)	51.6 (13.2)	49.1 (19.3)	44.9 (11.9)	0.17	−0.01	0.92
Oral reading recognition test (*n* = 51)	47.3 (9.1)	0.79	47.3 (8.6)	47.3 (9.4)	48.1 (10)	48.5 (7.5)	45 (10.5)	1.00	0.06	0.70
List sorting working memory test (*n* = 51)	49.9 (6.6)	0.99	51.6 (7)	49.3 (6.5)	49.5 (5.4)	49.3 (8.2)	49 (7)	0.31	0.13	0.37
Flanker inhibitory control and attention test (*n* = 51)	40 (6.3)	0.32	40.3 (5.4)	39.9 (6.7)	39.2 (6.5)	40.5 (8.4)	40.4 (5.6)	0.81	−0.07	0.65
Dimensional change card sort test (*n* = 51)	49.7 (12.4)	0.98	46.5 (11.9)	50.8 (12.5)	50.6 (13.7)	50.7 (10.4)	51.4 (13.3)	0.28	0.12	0.40
MRI‐derived functional/structural brain metrics										
White matter volume (*n* = 45) unit = mm^3^	398,226.7 (42,312.1)	NA	390,658.4 (54,525.4)	400,978.8 (37,555.4)	389,942.3 (36,884.9)	422,613.9 (49,610.9)	403,492.6 (22,778.2)	0.55	−0.11	0.49
Cortical thickness (*n* = 45) unit = mm	4.7 (0.2)	NA	4.7 (0.2)	4.7 (0.2)	4.7 (0.2)	4.6 (0.3)	4.7 (0.1)	0.32	−0.24	0.12
Gray matter volume (*n* = 45) unit = mm^3^	500,321.5 (33,907.2)	NA	503,221.1 (49,734)	499,267.1 (26,949.5)	499,216 (21,066.6)	501,952.3 (35,450.1)	497,469.4 (31,553.7)	0.80	−0.32	0.03
Subcortical gray matter volume (*n* = 45) unit = mm^3^	48,136.2 (3731)	NA	46,933.3 (5091.6)	48,573.6 (3080.5)	47,964 (2991.7)	49,642.4 (3504.6)	48,800.8 (3004.7)	0.31	−0.39	0.01
Total white matter hyperintensities volume (*n* = 45), (unit = mm^3^)	1788.3 (2381.3)	NA	3211.1 (3349.6)	1270.9 (1704.5)	1458.9 (2357.5)	1536.2 (877.3)	784.6 (416.3)	0.08	0.24	0.11
Global mean diffusivity (*n* = 42), unit = mm^2^/s	0.00066 (0.00002)	NA	0.00067 (0.00002)	0.00065 (0.00002)	0.00065 (0.00002)	0.00064 (0.00002)	0.00066 (0.00002)	0.10	0.22	0.16
Global fractional anisotropy (*n* = 42)	0.47 (0.02)	NA	0.46 (0.02)	0.47 (0.02)	0.47 (0.02)	0.48 (0.01)	0.47 (0.02)	0.03	−0.22	0.16
Median cerebral blood flow (*n* = 39), unit = mL of blood per minute per 100 g of brain tissue	1.06 (0.14)	NA	1.05 (0.1)	1.06 (0.15)	1.04 (0.12)	1.14 (0.25)	1.06 (0.14)	0.72	−0.28	0.09

Participants were categorized as nonobese (body mass index [BMI] < 30 kg/m^2^), obesity class I (30 kg/m^2^ ≤ BMI < 35 kg/m^2^), class II (35 kg/m^2^ ≤ BMI < 40 kg/m^2^), and class III (BMI ≥ 40 kg/m^2^). Available case analysis was used to manage missing values. Analyses were completed using R.v.4.1.1.

NA, not applicable.

Participants with obesity had higher fractional anisotropy (highly directional white matter microstructure) compared to individuals without obesity (*p* = 0.03). T2d duration correlated with smaller gray matter (*r* = −0.32, *p* = 0.03) and subcortical gray matter volumes (*r* = −0.39, *p* = 0.01) (Figure 1).

Regression revealed that longer T2d duration associated with smaller mean cortical thickness, gray matter volume, subcortical gray matter volume, and larger WMH volume, adjusted for total intracranial volume (Table 3). Additionally, higher systolic blood pressure associated with increased mean diffusivity.

**Table 3:** Association between cognition, MRI‐derived structural and functional morphology, metabolic risk factors and microvascular diabetes complications.

Variable	NIH toolbox composite	White matter volume (unit = mm^3^)	Mean cortical thickness (unit = mm)	Gray matter volume (unit = mm^3^)	Subcortical gray matter volume (unit = mm^3^)	Log (total white matter hyperintensities volume) (unit = mm^3^)	1000* global fractional anisotropy	1,000,000* global mean diffusivity (unit = mm^2^/s)	1000* median cerebral blood flow (unit = mL of blood per minute per 100 g of brain tissue)
	PE (95% CI)	PE (95% CI)	PE (95% CI)	PE (95% CI)	PE (95% CI)	PE (95% CI)	PE (95% CI)	PE (95% CI)	PE (95% CI)
Metabolic risk factors									
T2d duration study entry (years)	−0.01 (−0.32, 0.3)	46.49 (−576.41, 669.38)	−0.0061 (−0.0113, −0.0009)*	−830.39 (−1503.14, −157.64)*	−123.88 (−203.88, −43.89)*	0.0389 (0.0049, 0.0729)*	−0.42 (−1, 0.17)	0.6 (−0.15, 1.34)	−4.32 (−9.36, 0.72)
BMI (kg/m^2^)	0.16 (−0.21, 0.53)	65.74 (−689.09, 820.58)	−0.0025 (−0.0091, 0.0042)	−614.49 (−1466.76, 237.78)	54.54 (−51.74, 160.82)	−0.0355 (−0.0779, 0.0068)	0.57 (−0.13, 1.27)	−0.37 (−1.29, 0.54)	−0.37 (−6.3, 5.56)
Number of MetS (in addition to diabetes)	0.55 (−2.15, 3.25)	1052.37 (−4212.56, 6317.31)	−0.02 (−0.0664, 0.0263)	−5663.7 (−11,505.34, 177.94)	82.72 (−668.72, 834.16)	−0.1113 (−0.4151, 0.1924)	1.53 (−3.57, 6.63)	−0.42 (−6.97, 6.13)	9.77 (−34.52, 54.05)
SBP (mmHg)	−0.1 (−0.27, 0.07)	−16.01 (−413.22, 381.2)	−0.0012 (−0.0047, 0.0023)			0.0004 (−0.0226, 0.0234)	−0.32 (−0.69, 0.05)	0.61 (0.17, 1.06)*	−1.79 (−4.86, 1.29)
HbA1c (%)	−0.54 (−1.75, 0.67)	1086.98 (−1305.56, 3479.53)	−0.0033 (−0.0247, 0.0181)	−1184.76 (−3955.47, 1585.95)	−134.68 (−476.53, 207.17)	0.0517 (−0.0874, 0.1908)	−1.08 (−3.37, 1.21)	0.32 (−2.65, 3.28)	16.55 (−2.04, 35.15)
Triglycerides (mg/dL)	0 (−0.03, 0.02)	21.38 (−28.35, 71.11)	−0.0001 (−0.0005, 0.0004)	−19.07 (−76.8, 38.66)	0.31 (−6.84, 7.46)	0.0016 (−0.0013, 0.0045)	0 (−0.04, 0.05)	0.01 (−0.05, 0.07)	0.23 (−0.15, 0.61)
HDL (mg/dL)	−0.13 (−0.42, 0.15)	−282.96 (−839.42, 273.5)	0.0001 (−0.0049, 0.0051)	186.22 (−462.9, 835.35)	20.88 (−59.16, 100.92)	−0.0089 (−0.0415, 0.0236)	−0.14 (−0.68, 0.4)	0.09 (−0.6, 0.78)	0.6 (−4.31, 5.51)
Microvascular complications of diabetes									
MNSI index	−0.66 (−2.55, 1.23)	1665.22 (−2087.12, 5417.56)	0.0074 (−0.0261, 0.0409)	−1106.81 (−5475.05, 3261.44)	−297.28 (−829.21, 234.64)	0.203 (−0.0073, 0.4132)	2.07 (−1.71, 5.85)	−0.37 (−5.28, 4.54)	7.19 (−23.59, 37.98)
E/I ratio (unit = 0.05)	0.14 (−1.19, 1.46)	175.71 (−2357.87, 2709.3)	0.0079 (−0.0144, 0.0303)	2262.72 (−582.78, 5108.23)*	383.96 (43.11, 724.8)*	−0.1637 (−0.3014, −0.0259)	0.58 (−1.82, 2.98)	−2.91 (−5.84, 0.02)	−1.18 (−20.68, 18.31)
GFR	0.02 (−0.04, 0.08)	24.06 (−104.28, 152.39)	−0.0003 (−0.0014, 0.0009)	−27.22 (−175.69, 121.25)*	21.97 (4.97, 38.97)*	−0.0082 (−0.0152, −0.0012)	0.03 (−0.1, 0.15)	−0.06 (−0.23, 0.1)	1.12 (0.1, 2.14)*

Each row represents a single model. NIHTB‐CB is standardized based on participant age, sex, race, and education level. MRI‐derived functional/structural brain metric models adjusted for estimated total intracranial volume. We determined the number of metabolic syndrome components using the 2009 harmonized criteria. Available case analysis was used to manage missing values. Analyses were completed using R.v.4.1.1.

BMI, body mass index; CI, confidence interval; E/I ratio, expiration/inspiration ratio; GFR, glomerular filtration rate; HbA1c, hemoglobin A1c; HDL, high density lipoproteins; MetS, metabolic syndrome; MNSI, Michigan Neuropathy Screening Instrument; PE, point estimate; SBP, systolic blood pressure.

* Indicates statistical significance (*p* < 0.05) based on a two‐sided *p*‐value.

Lower E/I ratio and GFR associated with decreased subcortical gray matter volume and increased WMH (Table 3). Higher GFR is also associated with increased cerebral blood flow. Neuropathy did not associate with any outcome.

## Discussion

We found that cognition in a middle‐aged cohort of Pima American Indians with longstanding T2d did not differ significantly compared to demographic‐adjusted normative data. We found that T2d duration associated with decreased mean cortical thickness, gray matter volumes, and increased WMH. Otherwise, other metabolic risk factors, including obesity, did not consistently associate with cognition or MRI‐derived functional/structural brain outcomes. Lastly, we found that CAN and CKD, but not neuropathy, associated with decreased gray matter volume and increased WMH volume, indicating a potential correlation to cognitive disorders.

Meta‐analyses have found an increased risk of cognitive decline and dementia for individuals with diabetes and midlife obesity across multiple diverse populations.^4^, ^5^ However, we found that only T2d duration associated with impaired functional/structural brain metrics, suggesting obesity has secondary importance in this population with longstanding diabetes. This contrasts with our previous study of patients with obesity class II/III (mean (SD) age: 44.1 (12.1) years) which found obesity, not T2d, associated with poorer cognition, as measured by the NIHTB‐CB.^16^ The observed associations in the Pima American Indians, are comparable to studies comprised of older adults (mean ages: 65.0^17^ and 69.6^18^ years), which found that obesity was less of a risk factor than T2d for cognitive impairment.^17^, ^18^ Therefore, one possible explanation for findings in the present study is that Pima American Indians had longer T2d duration at a given age than other race/ethnic groups, possibly amplifying the effect of T2d as a risk factor for cognition relative to obesity. On the other hand, a recent longitudinal study of 403 American Indians found neither BMI or T2d associated with change in cognitive function over a 7‐year period, suggesting these factors might have less importance in this population.^19^ Currently, the underlying mechanisms linking cognition with both T2d, and obesity are not well understood. However, results from studies that measured the relative effects of obesity and T2d on cognitive impairment,^17^, ^18^ along with the results from the present study indicate that although obesity and T2d are often comorbid, there are likely independent underlying pathophysiologic mechanisms linking these risk factors with cognition.

We found that CAN and CKD measures associated with smaller gray matter volumes and increased WMH, consistent with our recent study based on a large cohort of individuals with and without T2d, which found that diabetes complications increased the odds of a cognitive disorder by 2.45 in those aged 40–60 years.^6^ Our results are also consistent with a systematic review that found cognitive impairment was common in individuals with CKD,^20^ although in a separate study of American Indians of the Zuni Pueblo, CKD measurements did not associate with cognition.^21^ Furthermore, a single study found heart rate variability CAN outcomes, associated with cognition.^22^ In contrast, we were surprised neuropathy did not associate with cognitive function, despite a previously documented relationship,^6^ possibly from a lack of statistical power in the present study. The underlying mechanisms dictating the relationship between cognitive impairment, CAN, and CKD remains unknown. Poorer CAN/CKD might directly impair or alternatively be comorbid with functional/structural brain metrics. Regardless of underlying mechanisms, our study indicates that preventing CAN and CKD in individuals with T2d might exert beneficial brain health consequences. Therefore, our study highlights the importance of screening CAN and/or CKD patients for early signs of cognitive impairment or dementia.

Our study limitations include small sample size, lack of control group without diabetes, cross‐sectional design, and unclear generalizability to other populations.

In this relatively small cohort of Pima American Indians with T2d, we found diabetes duration, but not obesity or other metabolic risk factors, associated with poorer cognition, smaller mean cortical thickness, and gray matter volumes, and higher WMH. Additionally, CAN and CKD correlated with worse functional/structural brain metrics, indicating the importance of preventing these complications for patients with T2d. In conclusion, our study highlights the importance of screening for cognitive disorders in individuals with longstanding T2d, particularly individuals with CAN and CKD, to provide the best care for patients with these debilitating conditions.

## Author Contributions

Dr. Reynolds was involved in the study design, performance, and interpretation of the statistical analysis, and wrote the manuscript. Dr. Votruba was involved in the interpretation of the data, and critical revisions of the manuscript. Dr. Jack was involved in the interpretation of the data, and critical revisions of the manuscript. Dr. Beare was involved in the interpretation of the data, and critical revisions of the manuscript. Dr. Reid was involved in the interpretation of the data, and critical revisions of the manuscript. Mr. Preboske was involved in the interpretation of the data, and critical revisions of the manuscript. Ms. Waseta was involved in the interpretation of the data, and critical revisions of the manuscript. Dr. Pop‐Busui was involved in the interpretation of the data, and critical revisions of the manuscript. Dr. Nelson was integrally involved in the study design, interpretation of the data, and critical revisions of the manuscript. Dr. Callaghan was involved in the study design, interpretation of the statistical analysis, and critical revisions of the manuscript. Dr. Feldman was integrally involved in obtaining the study funding, training the research coordinators, the study design, interpretation of the data, and critical revisions of the manuscript.

## Funding Information

Dr. Reynolds is supported by the NIH NIDDK (K99DK129785). Dr. Jack is supported by the NIH and the Alexander Family Professorship at Mayo Clinic. Ms. Waseta is supported by The Intramural Research Program of the National Institute for Diabetes, Digestive and Kidney Disease. Dr. Pop‐Busui is supported is supported by the NIH NIDDK (R01DK107956, U01DK119083, 1U01 DK0945157, R01DK116723) and the JDRF Center of Excellence at the University of Michigan. Dr. Nelson is supported by The Intramural Research Program of the National Institute for Diabetes, Digestive and Kidney Disease. Dr. Callaghan is supported by the NIH NIDDK (R01DK115687). Dr. Feldman is supported by the NIH (U01AG057562, U24DK115255, R01DK130913), the Robert E. Nederlander Sr. Program for Alzheimer's Research, the Andrea and Lawrence A. Wolfe Brain Health Initiative Fund, the A. Alfred Taubman Medical Research Institute, and the NeuroNetwork for Emerging Therapies.

## Conflict of Interest

Dr. Reynolds reports no disclosures. Dr. Votruba reports no disclosures. Dr Jack has consulted and served on data safety and monitoring boards but receives no financial compensation from any commercial entity. Dr. Beare reports no disclosures. Dr. Reid reports no disclosures. Mr. Preboske reports no disclosures. Ms. Waseta reports no disclosures. Dr. Pop‐Busui receives research support from Novo Nordisk. Dr. Nelson reports no disclosures. Dr. Callaghan consults for DynaMed, receives research support from the American Academy of Neurology and performs medical legal consultations including consultations for the Vaccine Injury Compensation Program. Dr. Feldman reports no disclosures.

## Supporting information


Data S1

## Data Availability

Dr. Reynolds is the guarantor of this work and, as such, had full access to all the data in the study and takes responsibility for the integrity of the data and the accuracy of the data analysis.
