# Supplementary material for: Association between brain health outcomes and metabolic risk factors in persons with diabetes
Source: Ann Clin Transl Neurol. 2023 Jul 30;10(10):1891–8. doi: 10.1002/acn3.51859 (PMC10578900; doi:10.1002/acn3.51859)
Supplement: Supplementary file 1 — Data S1 [file ACN3-10-1891-s001.docx]

Supplemental File:

*Details of MRI acquisition:* The MRIs were acquired on a single Philips (Amsterdam, Netherlands) 3T Ingenia scanner, using a protocol that included a 3D MPRAGE T1 weighted scan with 1.0mm^3^ isotropic resolution, a 3D pCASL with time to echo (TE)/repetition time (TR) 9/4050ms and 2.5x2.5x4.0mm^3^ voxels, and an axial 2D diffusion MRI with TE/TR 86/2363ms, (2.0mm)^3^ isotropic voxels and 12 b = 0 s/mm^2^ , 6 b = 500 s/mm^2^, 48 b = 1000 s/mm^2^, and 60 b = 2000 s/mm^2^ volumes, all isotropically distributed over spherical shells using an electrostatic repulsion scheme^23^. The protocol also included an axial 2D T2* weighted scan with TE/TR 20/650ms and 0.8x0.8 x4.0mm^3^ voxels, and a 3D Time-Of-Flight angiography of the Circle of Willis with TE/TR 2.533/30ms and 0.39x0.39x1.6mm^3^ voxels.
